# The interplay among familial risk, narrative coherence, and emotional problems from early to middle childhood

**DOI:** 10.3389/fpsyg.2023.969974

**Published:** 2023-02-15

**Authors:** Fabio Sticca, Olivia Gasser-Haas, Corina Wustmann Seiler

**Affiliations:** ^1^Research Department, Marie Meierhofer Children’s Institute, Zurich, Switzerland; ^2^Institute for Educational Support for Behaviour, Social-Emotional, and Psychomotor Development, University of Teacher Education in Special Needs, Zurich, Switzerland; ^3^Department of Pre-Primary and Lower Primary Level, Zurich University of Teacher Education, Zurich, Switzerland

**Keywords:** familial risk factors, emotional problems, narrative coherence, early childhood, resilience

## Abstract

The present study is a longitudinal extension (long-term follow-up) of a previous study examining the promotive and protective role of children’s narrative coherence in the association between early familial risk factors and children’s emotional problems from early to middle childhood. A total of 293 (T1; *M*_age_ = 2.81), 239 (T2; *M*_age_ = 3.76), and 189 (long-term follow-up T3; *M*_age_ = 9.69) children from 25 childcare centers participated in this study. Familial risk factors were assessed at T1 using a caregiver interview and questionnaire. Narrative coherence was assessed using the MacArthur Story Stem Battery that was administered to the children at T2. Children’s emotional problems were rated by the caregivers and by their teachers at T2 and T3. Results suggest that familial risk factors are linked to more emotional problems both in the short-term (T2) and the long-term (T3). Further, although some of the effects of relevant magnitude did not reach statistical significance, results pertaining to the role of narrative coherence indicate that it might have a promotive and protective effect in the short-term as well as a promotive effect in the long-term. These findings point to the relevance of children’s narrative coherence as a cognitive ability and personality factor that contributes to more positive development and to better coping with adverse familial experiences.

## 1. Introduction

Early childhood is a time of unparalleled development. During the first 3 years of life, most children develop from the state of a newborn that is dependent on their caregivers’ care even in the most basic of tasks, to a child that can or begins to show highly complex behaviors: They acquire fine and gross motor skills (Adolph and Berger, 2011), they begin to master one or more languages (Plunkett, 1997), and they start to understand physical laws (Wilkening and Cacchione, 2011), and other’s thoughts and feelings (Wellman et al., 2001). In short, they learn how to interact with their physical and non-physical environment. While the foundation of healthy ontogenetic development can be consolidated during the first years of life, this fundament can also be compromised by adverse childhood experiences (Liming and Grube, 2018), both inside and outside the familial context. Within a resilience framework (Masten and Barnes, 2018), the aim of the present study is to longitudinally extend results obtained by Müller et al. (2014) by examining if narrative coherence protects children from undesirable effects of early familial risk factors from early to middle childhood.

The familial context is central during the first years of life (Masten and Shaffer, 2006). On the one hand, a large body of research has identified various aspects of the family environment that can put children at risk in terms of their development in virtually all developmental domains (Sameroff and Seifer, 1983; Masarik and Conger, 2017). On the other hand, there are various factors that can compensate for the effects of familial risk factors (i.e., promotive effects) or even buffer their undesirable effects (i.e., protective effects; Benzies and Mychasiuk, 2009). In other words, not all children respond to early familial risk with maladaptive development. These promotive and protective factors can be found on various levels of a child’s environment and/or within the child itself (Masten and Barnes, 2018). Thus, it is crucial to understand the interindividual differences in children’s responses to familial risk factors. This knowledge is of pivotal importance for the development of tailored interventions aimed at directly reducing the occurrence of familial risk but also at reducing their effects (Chmitorz et al., 2018).

One key individual characteristic that has been found to protect from the detrimental effects of familial risk factors, is narrative coherence (Adler et al., 2018). Reese et al. (2011, p. 425) proposed a working definition of narrative coherence: “A coherent personal narrative is one that makes sense to a naïve listener – not just in terms of understanding when, where, and what event took place, but also with respect to understanding the meaning of that event to the narrator.” Narrative coherence has been conceptualized as the ability of an individual to process and organize his/her experiences into an internal working model (Bretherton, 1990; Oppenheim and Waters, 1995). Herein, an internal working model can be understood as a complex set of cognitive and emotional mental representations about the self, others, and interpersonal relationships. These internal working models are assumed to guide behavior through aspects of information processing such as attention, memory, and interpretation (Pietromonaco and Barrett, 2000). Narrative performance progressively develops from simple narrations to complex ones during middle childhood and becomes an aspect of an individual’s personality (Waters et al., 2019) that is positively linked to a series of desirable outcomes across the lifespan such as psychological wellbeing and identity functioning (Waters and Fivush, 2015; Vanderveren et al., 2021).

A central aspect of children’s wellbeing is emotional wellbeing. Given that emotional problems such as anxiety and depression are important public health issues (Costello et al., 2003; Choi, 2018), the present study focused on the interplay between familial risk factors, narrative coherence, and emotional problems from early to middle childhood. In the context of early childhood, only a few studies have addressed this topic. Müller et al. (2014) were able to show that children with higher scores of narrative coherence at age 3–5 years have lower scores of internalizing problems (i.e., promotive effect) and suffer less from the effects of familial risk factors on both internalizing and externalizing symptoms (i.e., protective effect). In a similar study, Stadelmann et al. (2015) reported that children’s narrative coherence mitigated the effects of maternal stress on their internalizing problems, although no promotive effect of narrative coherence was found. These associations might be explained by an increased capacity of the child to process information (Stadelmann et al., 2015), both of negative and positive nature. While these results point to a promotive and/or protective effect of narrative coherence, the robustness of these findings must be examined in further studies. Moreover, there has yet to be a study that examines the longitudinal role of narrative coherence in the association between early familial risk factors and children’s emotional development.

The present study aimed to expand our knowledge about the longitudinal role of narrative coherence in the interplay between early familial risks and children’s emotional development from early to middle childhood. The study is a longitudinal extension of a previous cross-sectional study that was published by Müller et al. (2014) for the period of early childhood. The following hypotheses were constructed for the present study based on the short-term pattern of results described above:

*H1*: Early familial risks are positively associated with emotional problems in middle childhood.

*H2*: Narrative coherence in early childhood is negatively associated with emotional problems in middle childhood.

*H3*: Narrative coherence in early childhood buffers the effect of early familial risks on emotional problems in middle childhood.

## 2. Methods

### 2.1. Sample and procedure

Twenty-five childcare centers were recruited from different cantons of German-speaking Switzerland. All 63 childcare center groups were included. A total of three waves of data assessment were realized. The first assessment (T1) was done in 2009 and included 293 children (*M*_age_ = 2.81; SD_age_ = 0.55; 47.9% female) and their primary caregivers. In 2010, a sample of 239 children (*M*_age_ = 3.76; SD_age_ = 0.49; 47.3% female) agreed to continue their participation in the second wave of data collection (T2; participation rate = 81.5%). Finally, a long-term follow-up was carried out in 2016 (T3) and 189 children (*M*_age_ = 9.69; SD_age_ = 0.48; 48.6% female) and their primary caregivers agreed to participate again (participation rate = 65% of the T1 sample and 79% of the T2 subsample). The sample was well educated, with 63.1, 64.8, and 69.5% of primary caregivers with at least a university degree in T1, T2, and T3, respectively. As for nationality, 89.2% of the primary caregivers were Swiss. Only 15.8% of the primary caregivers reported not speaking German in their homes. This study was not preregistered.

### 2.2. Ethical procedure

The primary caregivers were informed about the objectives and the procedure of the study and gave their written consent to the described procedure in a project description that was handed out beforehand. All primary caregivers and their children were also informed about their right to discontinue their participation at any time without indicating any reasons. Further, primary caregivers were informed that all data would be stored on a secure server at the Marie Meierhofer Children’s Institute in anonymized form and will be used exclusively for research purposes. Children received a small gift after each assessment. The study procedures were in accordance with Swiss legislation. The Cantonal Ethics Committee confirmed that no ethical approval was required for this specific project and its procedures.

### 2.3. Study measures

#### 2.3.1. Familial risk factors

The strategy for the assessment of familial risk factors as well as a discussion of its advantages and disadvantages was described in detail in previous publications from this study (Müller et al., 2014; Sticca et al., 2020). In short, familial risk factors were assessed at T1 using a combination of a paper and pencil questionnaire and a personal interview to reduce the effect of social desirability. Questions were adapted from instruments previously used in research on familial risk factors (e.g., Rutter & Quinton, 1977; Esser, 1989). In line with the cumulative risk hypothesis (for review see Evans et al., 2013), we aimed to assess a cumulative score of multiple risk factors tapping into a broad spectrum of risks. Specifically, 14 familial risk factors were included in the cumulative familial risk score. The operationalization of all risk factors together with the respective cut-off criteria for the presence of the risk factor as well as the resulting frequencies can be found in Table 1. The mean score of these 14 dichotomous or dichotomized familial risk factors was calculated to obtain an overall familial risk score.

**Table 1:** Operationalization of all indicators of familial risk factors.

Indicator of familial risk	%	Type	Condition for a code of 1 (i.e., child is exposed the respective risk factor)
Single parent family	10.0	Dich.	M or F reports being single.
Alcohol and/or drug abuse of F and M	5.4	Dich.	M and F report using alcohol and/or other drugs.
Current and/or previous family violence	3.4	Dich.	M and/or F reports that the child witnessed violence between caregivers.
Current and/or previous chronic partnership disharmony	10.7	Dich.	M and/or F reports that the child witnessed long lasting verbal conflicts between caregivers.
Family income below poverty threshold	12.7	Ord.	M and F report that their household income is below the poverty threshold of CHF 5200 per month.
Low maternal education	7.9	Ord.	M reports having a highest academic degree of primary or secondary level.
Immigrant background of the family	16.8	Dich.	M and/or F reports that their family language differs from the local language.
Serious illness or death of a primary caregiver	3.0	Dich.	M and/or F reports that a primary caregiver died or suffered from a serious illness in the last 12 months.
Serious illness or death of another family member	2.7	Dich.	M and/or F reports that a family member other than a primary caregiver died or suffered from a serious illness in the last 12 months.
Serious illness or death of a friend	1.0	Dich.	M and/or F reports that a child’s close friend died or suffered from a serious illness in the last 12 months.
Serious illness of a sibling	4.1	Dich.	M and/or F reports that a sibling suffers from a serious illness.
Self-reported mental health issues of M and/or F	4.9	Dich.	M and/or F reports that she/he is not doing well in terms of subjective mental health.
Move of the family	25.0	Dich.	M and/or F reports that the family moved in the last 12 months.
Current or previous issues with the law	1.9	Dich.	M and/or F reports that at least one caregiver was accused, brought before the court, and/or has been incarcerated.
Mean score of familial risk factors	7.6	Cont.	Percentage of experienced familial risk factors.

M, Mother; F, Father; Dich., Dichotomous original variable; Ord., Ordinal original variable; Cont., Continuous original variable.

#### 2.3.2. Emotional problems

The primary caregivers *and* the teachers completed the *Strengths and Difficulties Questionnaire* (SDQ; Goodman, 1997) both T2 and T3. Herein, the rationale is that a mixed report from multiple informants results in a more holistic picture of a child’s *trait* of the construct of interest (Kraemer et al., 2003). This strategy has been applied in other studies using the SDQ (White et al., 2021). Only the subscale of emotional problems (five items) was used for the present analyzes. Each item was rated on a 3-point Likert scale from 1 (not true) to 3 (certainly true). The ratings of the primary caregivers and those of the teachers were averaged for each item separately and then used as multi-informant indicators for further analyzes. In line with previous results from a series of confirmatory factor analyzes with data from both T2 and T3 (Sticca et al., 2020), two items were dropped and the multi-informant averages of three items (1) “many worries, often seems worried,” (2) “often unhappy, down-hearted or tearful,” and (3) “nervous or clingy in new situations, easily loses confidence” were used to model emotional problems. The resulting model with two latent variables (T2, T3) and only longitudinal correlations fitted the data very well (*χ*^2^[5] = 11.13, *p* = 0.13, CFI = 0.98, RMSEA = 0.05, SRMR = 0.06). As for the reliability, McDonald’s omega values were 0.75 for T2 and 0.65 for T3. Descriptive statistics are shown in Table 2. Tests for measurement invariance (Sticca et al., 2020) showed that metric but not scalar invariance was given (see Table 3), thus allowing for the comparison of variances and covariances, but not of means and intercepts (Cheung and Rensvold, 2002). Therefore, no comparison of the mean values of the emotional problems at T2 and T3 was possible.

**Table 2:** Zero-order bivariate correlations among all study variables (*N* = 238).

		*M*	SD	ICC	1	2	3	4	5	6	7	8	9	10	11	12	13
1	EPa - T2	1.07	0.28	0.11	1												
2	EPb - T2	1.11	0.35	0.05	0.64^***^	1											
3	EPc - T2	1.55	0.63	0.07	0.42^***^	0.47^***^	1										
4	EPa - T3	1.27	0.50	0.03	0.27^**^	−0.03	0.19^*^	1									
5	EPb - T3	1.14	0.40	0.06	0.02	−0.06	0.07	0.52^***^	1								
6	EPc - T3	1.41	0.55	0.04	0.09	0.02	0.21^**^	0.35^**^	0.30^**^	1							
7	NCa - T2	2.95	0.78	0.12	−0.09	−0.11	−0.13^*^	−0.05	−0.10	−0.07	1						
8	NCb - T2	3.00	0.79	0.10	−0.15	−0.07	−0.11	−0.32^**^	−0.28^**^	−0.12	0.24^**^	1					
9	NCc - T2	2.92	0.74	0.08	−0.05	−0.11	−0.08	0.02	−0.08	−0.09	0.18^*^	0.32^***^	1				
10	Female	52%	N.A.	0.04	0.01	0.06	0.03	−0.08	0.01	0.09	0.07	−0.03	−0.10	1			
11	Age	33.80	6.36	0.27	0.08	−0.06	−0.01	0.09	0.01	0.06	0.10	0.13	0.18^*^	0.04	1		
12	Cognition	7.79	2.34	0.04	−0.13	−0.14	−0.19^**^	−0.18	−0.21^*^	−0.26^*^	0.05	0.12	0.11	−0.12	−0.02	1	
13	Language	8.47	2.35	0.13	−0.12	−0.25^**^	−0.18^*^	0.06	0.12^*^	−0.10	0.06	0.06	0.17^*^	−0.10	0.36^***^	0.32^***^	1
14	FRF - T1	0.08	0.08	0.04	0.23^**^	0.21^**^	0.18^**^	0.18	0.30^*^	0.07	−0.07	−0.09	−0.13^*^	−0.01	0.04	−0.19^*^	−0.11

EP, Emotional problems; NC, Narrative coherence; Cognition, Score in the categorization task; Language, Score in the expressive language task; Subscripts_a,b,c_, Subscripts for the different items of the various constructs; FRF, Familial risk factors; T1-T2-T3, Waves of data assessment; ICC, Intraclass correlation. ^*^p < 0.05; ^**^p < 0.01; ^***^p < 0.001.

**Table 3:** Model fit comparison of the three models for the examination of measurement invariance.

	*χ* ^2^	df	*p*	CFI	RMSEA	SRMR	∆*χ*^2^	∆df	*p*
Configural	6.65	5	0.247	0.99	0.04	0.04	–	–	–
Metric	11.13	7	0.133	0.98	0.05	0.06	4.42	2	0.109
Scalar	50.99	9	0.000	0.79	0.15	0.09	63.20	2	0.000

*χ*^2^, Satorra-Bentler scaled Chi-square; df, degrees of freedom; *p*, probability of type I error; CFI, Comparative Fit Index; RMSEA, Root Mean Square Error of Approximation; SRMR, Standardized Root Mean Square Residual; *∆*, Difference value.

#### 2.3.3. Narrative coherence

The *MacArthur Story Stem Battery* (MSSB; Bretherton and Oppenheim, 2003) was used to assess narrative coherence at T2 [details reported by Müller et al., (2014)]. A trained experimenter uses a predefined set of toy figures to enact the beginning of a story to the child. The story then progresses into a situation where there is a conflict of interests for one or more of the children in the story. At this point, the experimenter stops and asks the child to continue enacting the story (“show and tell me what is happening now”). The child is then free to act out the story as she/he wishes. Three different stories were enacted by each child and were videotaped (Müller et al., 2014). Narrative coherence was scored using an adaption (Stadelmann, 2006) of the scoring manual by Robinson et al. (2002). Scores of narrative coherence for each story were 0 = “no answer,” 1 = “narrative is fragmented and no reference to the story stem,” 2 = “conflict (mostly) not picked up or no solution or conditions changed so that conflict can be avoided or addressed conflict, but most of the story was incoherent,” 3 = “slightly simplified conditions of conflict or conflict taken, picked up a small part but incoherent,” 4 = “conflict addressed with no inconsistencies.” Two independent raters scored 20% of the ratings. Interrater agreement was found to be low to moderate with Kappa = 0.55. Accordingly, the three measurements of narrative coherence were used as indicators of a latent variable to extract the reliable portion of the variance of the three indicators.

#### 2.3.4. Covariates

Children’s sex and age were reported by their caregivers at T1. Given that continuing the narration of a story stem requires both cognitive and language skills, we controlled for these skills in our models. Children’s cognitive ability and expressive language ability were assessed at T1 using a categorization task (cognitive ability) and an expressive language task from the *Development Test 6–6* (Petermann et al., 2006).

### 2.4. Analysis strategy

Several structural equation models were used to investigate the present research questions. A discussion of the advantages of this modeling strategy (i.e., promotive and protective effects in the association between early familial risks and children’s outcomes) and of the modeling details can be found elsewhere (Sticca et al., 2020). All latent variables were modeled using the effect coding method (Little, 2013). In a first step, a latent variable for emotional problems at T2 and another latent variable for its counterpart at T3 were modeled. Correlations between identical pairs of items of the two measurement occasions were freely estimated and emotional problems at T3 were regressed to emotional problems at T2. In a second step, familial risk factors (T1) were added to the model as a manifest predictor of emotional problems at both T2 and T3. In a third step, narrative coherence (T2) was introduced as a latent predictor of emotional problems for both T2 and T3. In a fourth step, we used the orthogonalization method (Little, 2013) to model a latent interaction term between familial risk factors and narrative coherence and added it to the model as a third latent predictor of emotional problems at both T2 and T3. Finally, we added cognitive ability, expressive language ability, as well as the child’s sex and age to the model so that emotional problems at both T2 and T3 could be regressed onto these manifest control variables. All exogenous variables were allowed to correlate with each other. All models proved to fit the data well (see Table 4). Regarding the treatment of missing data, a series of *t*-tests showed that children who participated in both T1 and T2 had comparable family risk scores (*β* = −0.09; *p* = 0.19) to those that only participated in T1. However, lower scores of familial risk factors were found between children participating in T2 and T3 as opposed to those dropping out after T2 (*β* = −0.15; *p* = 0.02). These two groups also had comparable scores for emotional problems (*β* = −0.02; *p* = 0.08) and narrative coherence (*β* = 0.16; *p* = 0.12) at T2. Accordingly, the Full Information Maximum Likelihood (FIML) method was used (Schafer and Graham, 2002). As for the handling of the clustered nature of the data, the use of a multi-level model was not indicated due to a low participating child count in each of the 63 childcare groups. Nevertheless, given that the intraclass correlation coefficients (ICC) varied between 0.03 and 0.13 (see Table 1), we used the Huber-White Sandwich Estimator (Freedman, 2006) to correct for the dependencies among children in the same childcare group.

**Table 4:** Model fit indices of the structural equation models.

	*χ* ^2^	df	*p*	CFI	RMSEA	SRMR
Model without control variables	44.00	57	0.90	1.00	0.00	0.06
Model with control variables	73.47	89	0.88	1.00	0.01	0.07

*χ*^2^, Chi-square; df, degrees of freedom; *p*, probability of type I error; CFI, Comparative Fit Index; RMSEA, Root Mean Square Error of Approximation; SRMR, Standardized Root Mean Square Residual. Models with covariate include children’s sex and age as well as their score in the categorization and expressive language tasks as manifest covariates.

## 3. Results

**Figure 1:**
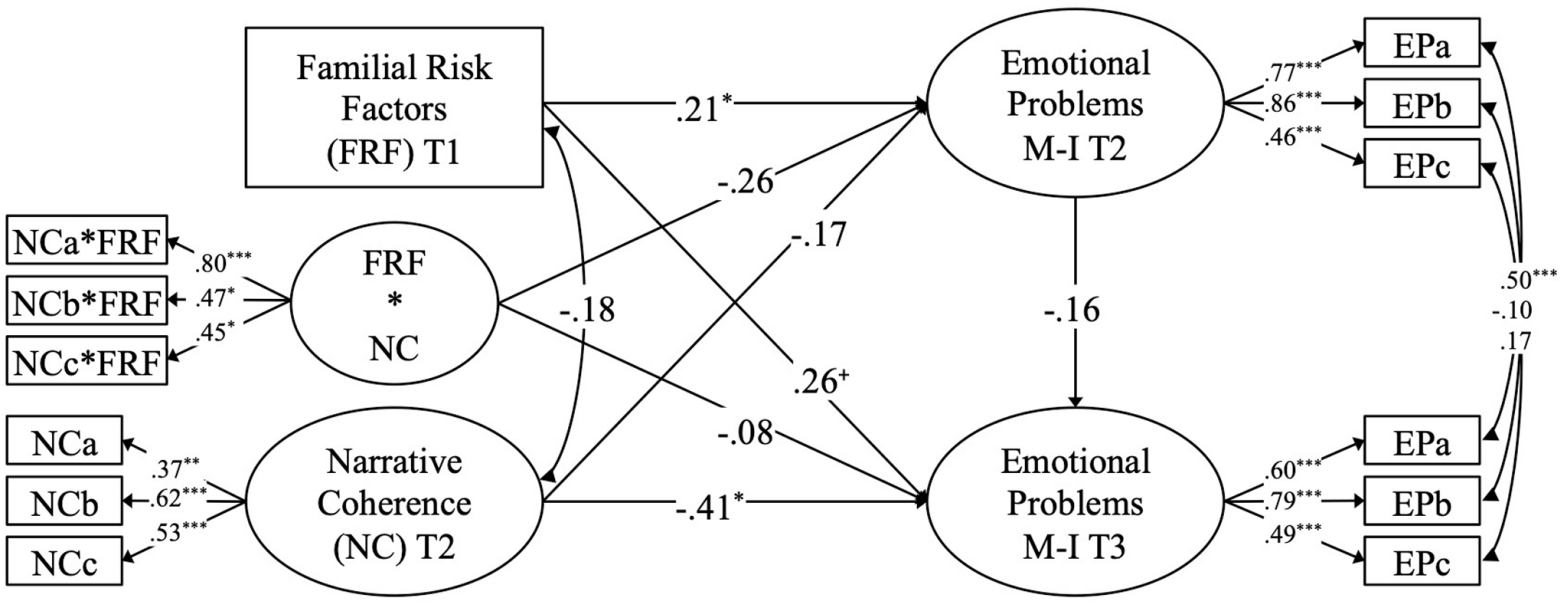
Standardized results from the model with narrative coherence (NC) as a moderator of the association between familial risk factors (FRF) and emotional problems (EP). FRF*NC represents interaction term between familial risk factors and narrative coherence. Control variables were omitted for simplicity. ^+^*p* < 0.10; ^*^p < 0.05; ^**^p < 0.01; ^***^p < 0.001.

Results from the structural equation model are displayed in Figure 1 and will be reported in the following starting with the results pertaining to the interaction between familial risk factors and narrative coherence in the prediction of emotional problems at T2, followed by results for the prediction of change in emotional problems from T2 to T3. Finally, additional results pertaining to the covariates will be reported.

### 3.1. Short-term results (early childhood)

A higher number of familial risk factors was significantly associated with higher scores in multi-informant emotional problems in early childhood, with a small-to-medium effect size. In contrast, higher scores on narrative coherence were linked to lower scores on emotional problems (i.e., promotive effect), although the small-to-medium effect did not reach statistical significance. Additionally, an indication of a protective effect was found in that higher scores on narrative coherence were linked to weaker associations between familial risk factors and emotional problems, although this effect did also not reach statistical significance. As for the control variables, no significant effects were found, although expressive language skills were found to show a small-to-weak negative effect on emotional problems (*β* = −0.15; *p* = 0.26).

### 3.2. Long-term results (early to middle childhood)

Emotional problems in middle childhood were found to be weakly but not significantly linked to emotional problems in middle childhood over and above all other predictors and control variables in the model. Further, familial risk factors were found to lead to more positive changes^1^ in emotional problems from early to middle childhood, with a medium effect size but only a marginal statistical significance. Moreover, higher scores on narrative coherence were found to lead to significantly more negative changes in emotional problems, with a medium-to-large effect size (i.e., longitudinal promotive effect). Regarding the protective effect, higher scores on narrative coherence were linked to more negative associations between familial risk factors and changes in emotional problems, although the effect was not found to be significant, and its size was comparably small. Turning to the control variables, only cognition was found to have a significant and medium-sized negative effect on changes in emotional problems (*β* = −0.27; *p* = 0.045). In contrast, language skills were found to have a non-significant positive effect on changes in emotional problems (*β* = 0.22; *p* = 0.18).

### 3.3. Additional results

Narrative coherence at T2 was found to have a marginally significant, small to moderate negative correlation to familial risk factors (*β* = −0.18, *p* = 0.09). Further, a marginally significant positive correlation between narrative coherence at T2 and cognitive (*β* = 0.20, *p* = 0.06) as well as linguistic skills (*β* = 0.18, *p* = 0.09) was found, with small to moderate effect sizes. While age was found to be positively correlated to narrative coherence at T2 (*β* = 0.25, *p* = 0.006), no gender difference was found (*β* = −0.06, *p* = 0.54). Additionally, familial risk factors were linked to lower cognitive ability (*β* = −0.19, *p* = 0.01) but not to language skills (*β* = −0.11, *p* = 0.12), gender (*β* = −0.01, *p* = 0.92), or age (*β* = 0.04, *p* = 0.61). Turning to the correlations among the covariates, children’s cognitive skills were positively correlated to their linguistic skills (*β* = 0.32, *p* < 0.001), and females were found to score marginally higher on cognitive skills as opposed to males (*β* = −0.13, *p* = 0.05), while no correlation to age was found (*β* = −0.02, *p* = 0.80). Moreover, linguistic skills were uncorrelated to gender (*β* = −0.10, *p* = 0.11), while older children reached significantly higher scores on linguistic skills (*β* = 0.36, *p* < 0.001). Finally, age and gender were not found to be correlated (*β* = 0.04, *p* = 0.58).

## 4. Discussion

The present study consisted in a longitudinal extension of a previous study on the role of children’s narrative coherence in the association between early familial risk factors and emotional problems in early childhood (Müller et al., 2014). Specifically, we investigated if narrative coherence longitudinally promotes adaptive emotional development and if it longitudinally protects against the undesirable effects of early familial risk factors from early childhood to middle childhood. Results will be discussed in the following, starting with familial risk factors and emotional problems followed by the promotive and protective role of narrative coherence.

### 4.1. Familial risk factors and emotional problems

Early familial risk factors were positively related to children’s emotional problems in the short-term. Therefore, hypothesis 1 can be confirmed. Results from the present study are in line with those pertaining to the association between familial risk factors and parent-reported emotional problems obtained by Müller et al. (2014). However, they are not in line with previous results on the link between familial risk factors and teacher-reported emotional problems, as the latter was not found to be significant in the previous study. Further, these results map onto those from another study stemming from the same project that only considered parent-reported children’s emotional problems (Sticca et al., 2020). Thus, results stemming from the multi-informant approach seem to align to those from the parent only but not to those from the teachers-only approach.

Turning to the long-term associations from early childhood to middle childhood, it must first be noted that emotional problems were found to have a very low relative stability. This might partly be explained by an actual fluctuation from early to middle childhood but also by a methodological factor: The informants on the teacher side changed from early to middle childhood, as children moved from childcare centers to primary school in the meantime. Another explanation for the lower stability in the multi-informant than in the parents-only approach (Sticca et al., 2020), is the inclusion of cognitive and linguistic covariates that might account for a certain degree of stability in emotional development.

Familial risk factors had a positive and marginally significant effect on emotional problems in middle childhood over and above emotional problems in early childhood (and all other covariates). This result is in line with those obtained by Sticca et al. (2020), although the magnitude of the effect is much higher (*β* = 0.26 vs. *β* = 0.10) and suggests that early familial risks might have long-term effects on children’s emotional development. The bivariate correlations suggest that this link is very different among the three indicators of emotional problems, although there is a general tendency for a positive trend. This pattern of results obtains a certain relevance if the time lag of 6 years between the assessments is considered. However, more research is needed to confirm the validity of this long-term result.

### 4.2. The role of narrative coherence

Children’s narrative coherence was found to have both a promotive and a protective short-term effect on emotional problems in early childhood, although both effects did not reach statistical significance despite having a small-to-medium effect size. Accordingly, hypotheses 2 and 3 must be rejected. This absence of statistical significance combined with the relevant magnitude of the effects suggest that the statistical power of the present analyzes was limited. This lack of power might also be attributed to a relatively low reliability of narrative coherence and to the manifest nature of the familial risk variable. Nonetheless, we would argue that these results point toward a possible short-term effect of narrative coherence: Children that are able to tell more coherent narratives in the context of a conflict-based story stem tend to be rated as lower on emotional problems by their caregivers and teachers. Further, these children seem to suffer less from the undesirable effects of early familial risks. These results are in line with those obtained by Müller et al. (2014) with respect to the caregiver ratings of emotional problems. Moreover, our results align with those reported by Stadelmann et al. (2015) as far as the protective effect is concerned, as the authors did not find evidence for a promotive effect. The difference regarding the promotive effect might lie in the broad/distal vs. narrow/proximal focus in the conceptualization of the risk factors (i.e., risk index vs. parental stress) and in a different approach to the modeling strategy (i.e., latent vs. manifest).

As for the longitudinal results, narrative coherence was found to have a significant medium-to-large promotive effect on emotional problems in middle childhood over and above emotional problems in early childhood. However, the corresponding long-term protective effect resulted as very small and non-significant. Given that this is the first study to address these research questions with a long-term perspective, the validity of these results remains to be confirmed in future studies. Thus far, we can tentatively derive from these results that narrative coherence seems to be beneficial for all children even in the long-term and to only have a minimal effect on reducing the long-term effects of early familial risks. This conclusion is in line with the concept of narrative coherence as the ability of an individual to build an internal working model of her/his environment (Bretherton, 1990; Oppenheim and Waters, 1995) and to self-regulate her/his behavior, cognitions, and emotions (Vallotton and Ayoub, 2011).

Overall, our results are in line with the assumptions of the resilience model (Masten and Barnes, 2018) as they point toward a complex interplay of family-based and child-based factors in determining short- and long-term outcomes on the child level. Specifically, we believe that our results map onto the concept of narrative coherence as an individual information processing skill that allows children to make sense of what they experienced (Bretherton, 1990; Oppenheim and Waters, 1995) and to adaptively interact with their environment (Müller et al., 2014; Stadelmann et al., 2015). Specifically, it might be assumed that children with a more developed narrative coherence are more likely to tell others about their experiences, to share their feelings, and thus to receive social support, which might in turn alleviate stress.

As far as implications for practice are concerned, our results imply that fostering children’s narrative coherence might be an avenue for both more adaptive emotional developments and better coping with familial risk factors. In line with suggestions by Masten and Barnes (2018) it is important to interpret this as one of a multitude of intervention pathways that must ideally be taken simultaneously. Such a combined intervention increases the chances of addressing various aspects at different levels of a system (i.e., child, family, community) and to unfold its potential for resilience.

### 4.3. Limitations

The present results must be interpreted in the light of the following limitations: Familial risk factors and children’s emotional problems were assessed using self-reports or other-reports and are thus subject to validity issues such as social desirability, selective perception, and memory effects. However, narrative coherence as well as cognitive and language skills were assessed using different methods thus counteracting the common methods bias. Another limitation is the suboptimal reliability of emotional problems and narrative coherence as well as the absence of scalar invariance in emotional problems. These results might have reduced the chances of finding the hypothesized effects. Further, familial risk factors were modeled without accounting for the severity of the various risk factors. In combination with the community-based sample of the present study, the interpretation of family risk factors must be made with caution. Finally, while the prolonged time window between assessments in this study is very valuable, it remains to be studied how the effects of familial risk factors and various promotive and protective factors unfold over time. This question needs to be addressed with more frequent assessments across childhood and thereafter. These conclusions notwithstanding, the unique end novel contributions of the present study enhance our understanding of the complex interplay between risk, promotive, and protective factors in early childhood.

## Data availability statement

The original contributions presented in the study are included in the supplementary material, further inquiries can be directed to the corresponding author/s.

## Ethics statement

Ethical review and approval were not required for the study on human participants in accordance with the local legislation and institutional requirements. Written informed consent to participate in this study was provided by the participants’ legal guardian/next of kin.

## Author contributions

FS was responsible for the data management, analyzed and interpreted the data, and wrote the first draft of the manuscript for the present study with valuable feedback from CWS and OG-H. CWS acquired the funding, designed, and managed the study, collected and developed the instruments, and recruited the sample. OG-H helped recruit the sample, organized the data collection, and collected the data together with several undergraduate students. FS, CWS, and OG-H have critically reviewed and revised the manuscript. All authors have read the final version of the manuscript and agreed to publish it in its present form.

## Funding

This study was supported by the Swiss National Science Foundation (Grant Nos. 100019_166003 and 100014_124949) and the Jacobs Foundation. The publication was funded by the Swiss National Science Foundation.

## Conflict of interest

The authors declare that the research was conducted in the absence of any commercial or financial relationships that could be construed as a potential conflict of interest.

## Publisher’s note

All claims expressed in this article are solely those of the authors and do not necessarily represent those of their affiliated organizations, or those of the publisher, the editors and the reviewers. Any product that may be evaluated in this article, or claim that may be made by its manufacturer, is not guaranteed or endorsed by the publisher.

## Footnotes

^1^The term “changes” refers to the score of emotional problems at T3 while controlling for scores of emotional problems at T2. This circumstance is due to the absence of scalar invariance and the resulting impossibility of modeling true change.
